# Understanding the value of brain donation for research to donors, next-of-kin and clinicians: A systematic review

**DOI:** 10.1371/journal.pone.0295438

**Published:** 2023-12-20

**Authors:** Cassandra P. Griffin, Jenna R. Bowen, Marjorie M. Walker, James Lynam, Christine L. Paul

**Affiliations:** 1 College of Health, Medicine and Wellbeing University of Newcastle, Tamworth, NSW, Australia; 2 Hunter Medical Research Institute, Newcastle, NSW, Australia; 3 Department of Medical Oncology, Calvary Mater, Newcastle, NSW, Australia; 4 Priority Research Centre Cancer Research, Innovation and Translation, University of Newcastle, Callaghan, Australia; 5 Priority Research Centre Health Behaviour, University of Newcastle, Callaghan, Australia; U.S. Food and Drug Administration, UNITED STATES

## Abstract

**Purpose:**

Post-mortem brain donation affords the opportunity to characterise disease by exploring global neuropathological changes. Such opportunities are essential to progress knowledge of CNS tumours such as Glioblastoma. A comprehensive understanding of the experience of consenting to brain donation is crucial to maximising consent rates while providing patient-centred care. This review aimed to synthesise the reported facilitators and barriers according to potential donors, next-of-kin (NOK) and clinician respondents.

**Design:**

Database searches included Embase, Medline, PsycINFO, Psychology and Behavioural Science and Scopus. Search terms focused on motivations, attitudes and psychosocial experiences of brain donation. Exclusions included organ transplantation and brain death. All studies were assessed for quality and validity using tools from the Joanna Briggs Institute. To determine perceptions of benefit and harm, a method guided by the thematic analysis of Braun and Clarke was employed to reflexively assess and identify common themes and experiences.

**Results:**

40 studies (15 qualitative, 25 quantitative) were included involving participants with paediatric cancer, neurodegenerative and psychological diseases. Perceptions of benefit included benefit to future generations, aiding scientific research, avoidance of waste, improved treatments and the belief that donation will bring consolation or aid in the grieving process. Perceptions of harm included a perceived conflict with religious beliefs, disfigurement to the donor, emotional distress at the time of autopsy and discord or objections within the family.

**Conclusion:**

Brain donation can afford a sense of purpose, meaning and empowerment for donors and their loved ones. Careful strategies are required to mitigate or reduce potential harms during the consent process.

## Introduction

High grade primary brain cancer such as Glioblastoma Multiforme (GBM) has a median survival of 13 months [1], with a distinct absence of clinical advancement over the last few decades [2]. Relatively little is known of the pathophysiology and neurobiology of primary brain cancers [3] and due to a number of unique clinical challenges such as targeted drug delivery through the blood brain barrier [4], the designation of glioma as an immunogenically ‘cold’ tumour [5] and vast intertumoral heterogeneity [4,6] the clinical trial pipeline is limited. The rise of personalised medicine and increase in tumour profiling has led to a renewed emphasis on understanding tumoral neurobiology. Therefore, the demand for high quality annotated biospecimens is increasing. Post-mortem brain donation affords researchers insight into spatial and temporal heterogeneity as well as ensuring a supply of tissue for histological, molecular and omics driven research platforms [7].

Optimising brain donation protocols to maximise the potential for psychosocial benefit and minimise the potential for harm or undue distress requires consent processes to be patient-centred and highly individualised. The diagnosis of brain cancer is associated with increased rates of depression and anxiety [8] and an overall reduction in psychosocial wellbeing [9] which places people in a potentially vulnerable state. In the absence of any immediate clinical hope, actions which can provide psychosocial benefit to patients and carers may be of value. Insights from work conducted in the transplantation space can provide an initial basis from which to explore this. From a carers perspective, the work of Riley *et al* suggests that participation in end of life decisions assists carers to overcome feelings of helplessness and powerlessness while also generating meaning from ‘senseless tragedy’ [10].

From a donor perspective, there is burgeoning literature base examining the motivations and obstacles to brain donation which is further complemented by qualitative studies exploring the experiences of parents in the paediatric neuro-oncology setting [11,12]. In addition, the transplantation literature suggests a potential psychosocial benefit for next of kin from organ donation [13]. Many of these studies were captured and assessed by Lin et al [14] in their systematic review assessing motivations for brain donation from the perspective of next of kin and potential donors. The review identified 4 key pillars that influence decision making namely contextual knowledge, conceptual understanding, personal experience and family/friends [14]. These pillars comprise a selection of the factors that can impact the outcome of decision making, or positively correlate with a completed donation such as health literacy [15], timeliness of the request [16] or communication with healthcare professionals [16].

What remains absent within the literature, however, is a systematic assessment of the perceptions of benefit and harm associated with brain donation protocols and how these perceptions may differ between participants including donors, next of kin and health care providers (HCPs).

Understanding the human experience of brain donation, using a broad pan-diagnosis literature search may provide a basis from which to better understand the experience of brain donation within a brain cancer setting. Such insights would provide a fundamental grounding from which further research into the psychosocial value of brain donation for people with brain cancer could be undertaken.

This review aims to:

- The assess the quality of the existing literature contributing to the field of knowledge
- To identify the potential harms and benefits of a post-mortem brain donation program
- To determine what variables may impact lived experiences of post-mortem brain donation programs for donors in terms of disease state, information and awareness, or culture

## Methods

### 1. Design and registration

A systematic review of the literature was conducted according to The Preferred Reporting Items for Systematic Reviews and Meta-Analyses (PRISMA) statement. The systematic review is a narrative synthesis of studies and registered with Prospero (International Prospective Register of Systematic Reviews–registration number CRD42021224467)

### 2. Literature search

Initial database searches commenced in September of 2020 and included Embase, Medline, PsycINFO, Psychology and Behavioural Science and Scopus. This was also supported by a search of the first 5 pages of Google scholar. The search was updated in January 2022 and August 2023.

The search was undertaken using keywords and medical subject heading searches (MesH) under the guidance of the university librarian. Boolean Operators “AND” and “OR” were used to combine search terms where appropriate, “OR” was used for within group combinations while “AND” was used for between group combinations. “NOT” was used to eliminate search terms. The search was restricted to studies with human participants and those published in English and employed the following search structure ((brain* N3 (donat* OR bank OR biobank*)) AND (psychological* OR griev* OR dying OR grief OR distress* OR attitude* OR Motivat* OR impact* OR “Informed consent*” OR decision*) AND (famil* OR “loved one” OR “next of kin” OR patient* OR person* OR individual* OR communit* OR donor* OR deceased* OR parent* OR child*). Given the overwhelming abundance of donation and transplantation literature, search exclusions included (NOT “transplantation”, “brain death”, “brain dead”, “renal” and “kidney”).

### 3. Inclusion criteria

- Study Design and type: cross-sectional quantitative studies and qualitative studies which included original data under the following categories:
  - Studies investigating the reactions, perceptions, or experiences with brain donation from the perspective of donors, relatives, next of kin or communities.
  - Studies exploring societal views of brain donation.
  - Studies investigating patterns of consent for brain donation and decision-maker motivations.
- Participants: Studies which documented the perspectives of donors (potential and actual), next of kin, health care practitioners and community members. Potential donors were defined as individuals who were discussing a theoretical participation in or interaction with a brain donation program. Brain donation programs were broadly included across disease types and not limited based on neuro-pathological focus or scientific/clinical question.

### 4. Exclusion criteria

Methods papers focusing primarily on refinement of biobanking protocols or recommendations for improved consent processes were excluded. Biobank case study papers documenting evolution of procedures were excluded as were papers conducting sentiment or thematic analysis on donors of regenerative tissues for transplantation.

### 5. Screening

All articles were exported to Endnote 20.3 and duplicates removed following the conclusion of the search process. After deduplication, articles were exported into Covidence for title and abstract screening. Title and abstract screening was completed independently by two reviewers (JRB, CPG) based on the inclusion and exclusion criteria. If the eligibility of the study could not be determined during the title and abstract screening it was included to allow for full-text screening. Full-text screening was completed independently by the same reviewers. Discrepancies were resolved between the two reviewers JRB and CPG with no need for a third reviewer. Reasons for exclusion during the full-text screening were recorded in Covidence.

### 6. Data extraction

Data extraction was performed independently by two members of the review team JRB and CPG. The reviewers discussed any discrepancies until resolved and did not require a third reviewer to resolve disagreements. The following data were extracted from each of the included studies:

- Publication details: author, study title, publication, publication year, country of study
- Study setting: disease type or purpose of brain banking program
- Sample size: including participant subgroup stratification if present
- Population: description of study population
- Study method: Surveys, telephone interviews, focus groups
- Subgroup: clinicians, potential donors, next of kin (bereaved or prior to donation)
- Findings/Results: perceptions of harm and perceptions of benefit

### 7. Quality appraisal

Quality appraisal was conducted by reviewers JRB and CPG using the suite of critical appraisal tools available through the Joanna Briggs Institute. All data types included in this analysis met either the criteria for the ‘Checklist for Analytical Cross-Sectional’ [17] data or ‘Checklist for Qualitative Research’ [18]. This process was carried out independently with two reviewers determining whether a study met or did not meet the eight criteria for the cross-sectional analysis or the ten criteria for qualitative research.

### 8. Data analysis and synthesis

A reflexive process, caried out by JRB and CPG was used to code and group extracted data. This process was guided by the principles of reflexive analysis as outlined by Braun and Clarke 2020 [19], though it should be noted a full thematic analysis was not employed due to the heterogeneity of the data and diverse methodological approaches. The process began with data familiarisation to emphasise context and setting, systematic data coding (in this case based on language repetition or language redundancy). The initial process involved a focus on identifying common language, themes and experiences to develop a concise picture of perceived harms and benefits shared by respondents throughout the literature. Subsequently the data were re-examined subjectively and with a deductive approach to identify recurring sentiments. This allowed data to be grouped and resulted in a set of codes. This ‘continuum’ approach to data synthesis allowed for an inclusion of the semantic to the latent within the data. Following analysis, data was collated into two tables for ease of interpretation. As per the methodology of Braun and Clarke, researcher subjectivity was employed as a resource for production, not a threat to credibility [19].

## Results

### Output from systematic search

**Fig 1:**
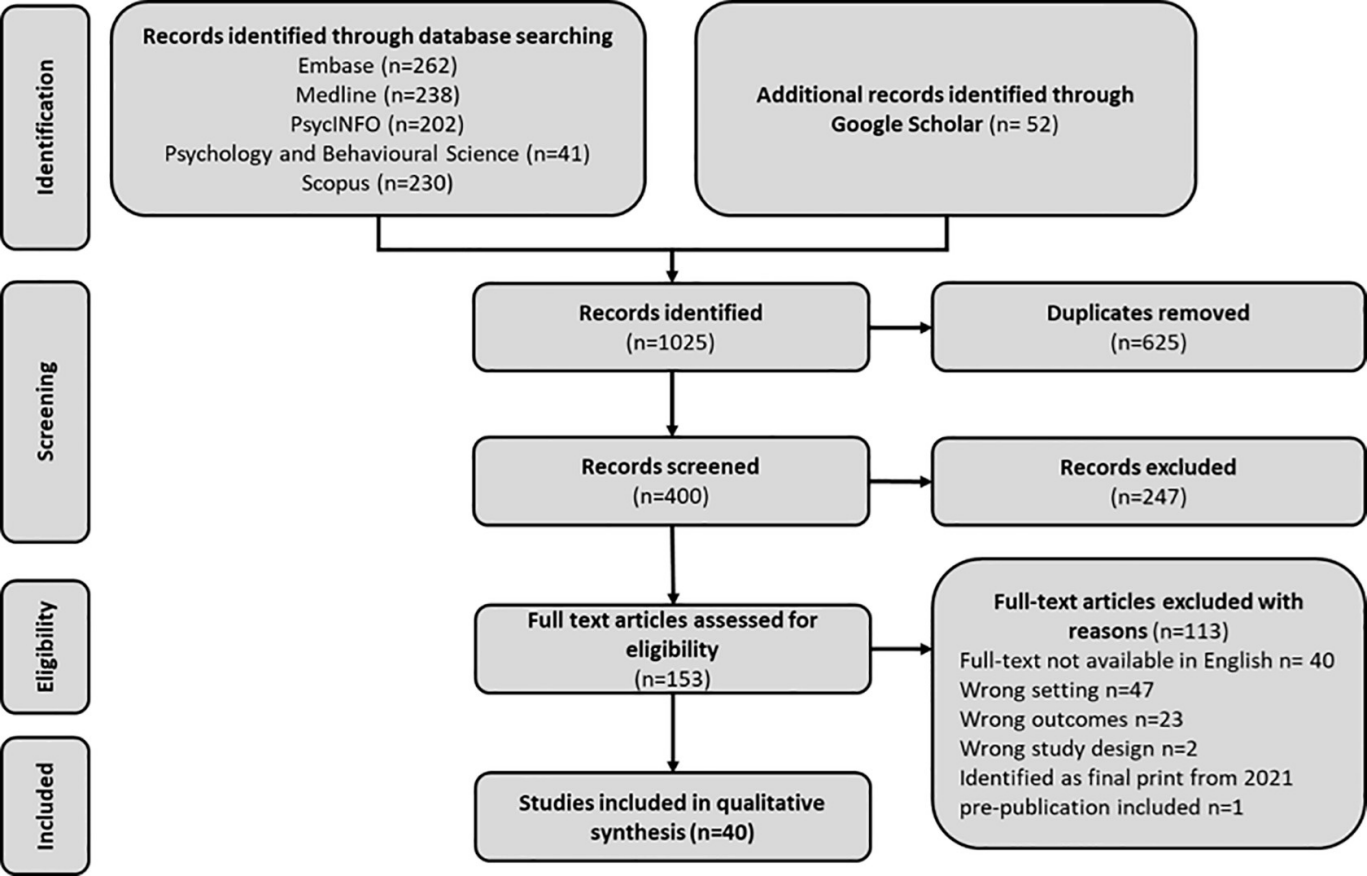
PRISMA guided search strategy. Fig 1 documents the search strategy employed, capturing both the 2020,2022 and 2023 search periods.

Fig 1 documents the results of the search and screening process, beginning with a total of 1025 papers and resulting in a final cohort of 40 (Fig 1).

Table 1 documents publication details, data type, study population, setting sample size and designated subgroup of participants for the 40 included studies.

**Table 1:** Included studies.

Lead author, Year and Country	Method	Data type	Population	Setting (i.e. disease)	Sample size	Subgroup
**Bidaut-Russell, 1991, USA [20]**	Written questionnaires	Cross-sectional	Next of Kin—designated on brain bank consent form	Senile dementia of the Alzheimer’s type (SDAT) 98.5% Caucasian	199	NOK (Including bereaved)
**Shaw, 2012, USA [21]**	Interviews carried out via phone and in person at various intervals.	Cross-sectional	Healthy participants in Georgia Centenarian Study. Mean age 100.6 yrs	Centenarians—otherwise healthy	66	Donors (consented)
					145	Potential donors (denied consent)
**Wu, 2012, Taiwan [22]**	Face to face questionnaires with 5-point scale		Outpatients, Dementia and incompetence excluded.	Mental illness (psychiatric Taipei hospital).	364	Potential donors
					210	NOK
**Darnell, 2011, USA [23]**	In home interviews	Cross-sectional	African American individuals, aged 65 or over	Normal controls for Alzheimer’s disease research	46	Potential donors
**Eatough, 2012, UK [16]**	Semi-structured interview with phenomenological informed theme analysis	Qualitative	Family members and friends of deceased London brain bank donors	Non-specific. UK brain banking network	19	Bereaved NOK
**Fonseca, 2015, Brazil [24]**	Phone and in person unstructured interviews	Cross-sectional	NOK of deceased	Neurodegenerative disease outpatients	30	Bereaved NOK
			Neurodegenerative disease Patients		14	Potential Donors
			NOK of potential participants		18	NOK
**Glaw, 2009, Australia [13]**	Written questionnaire	Cross Sectional, analytical study	Donors	Registered and consented brain donors from the NSW Tissue Resource centre	658	Donors (consented)
**Azizi, 2006, Australia [25]**	Telephone interview with descriptive statistics and them analysis	Cross-sectional	NOK of recently deceased individuals listed on the coroners list	NOK on the day of autopsy. Many of deceased with notable mental illness	48	Bereaved NOK
**Schnieders, 2013, USA [26]**	Semi-structured in-home interview. Sub-study within longitudinal aging study	Cross-sectional	African Americans (>65 years) invited to participate in an aging/Alzheimer’s study	Aging and Alzheimer’s research	61	Potential Donors
**Garrick, 2006 Australia [27]**	Web-based questionnaires—fixed and open ended.	Cross-sectional	Registered donors (pre consent)	Using our brains study (Healthy donors)	187	Donors
			Consented Donors			
**Kuhta, 2011, USA [28]**	30 item survey provided to consecutive patients	Cross-sectional	Consecutive patients presenting to movement disorders centre	Movement disorders	114	Donors (consented)
					36	Donors (not consented)
**Jefferson, 2011, USA [28]**	Posted written survey with follow up phone-calls. Data supported by existing registry demographic data	Cross-sectional	African American Elders consented to an Alzheimer’s research registry	Alzheimer’s disease registry participants (233)	49	Potential donors
			Caucasian Elders consented to an Alzheimer’s research registry		184	
**Millar, 2007, UK [29]**	Unstructured/informal interviews embedded in consent process	Qualitative	Next of kin following a sudden death	Normal brains or CNS disorder of interest	111	Bereaved NOK
**Le Bouc, 2016, France [30]**	Survey conducted across French memory centres—distributed by post and email	Cross-sectional	Consenting physicians	Dementia	42	Clinicians
						NOK (relayed by clinicians)
**Padoan, 2017, Brazil [31]**	Semi-structured interviews on a theoretically sampled population	Qualitative	12 with bipolar disorder and 6 family members	Bipolar disorder	18	Combined potential donor and NOK
**Angelini, 2011, Canada [32]**	Unstructured/informal interviews embedded in consent process	Cross-sectional	Parents/guardians of children diagnosed and treated with DIPG in a Canadian hospital.	DIPG	21	NOK (parents)
**Stevens, 1998, UK [33]**	Written questionnaire delivered in person or by post inclusive of MCQ and open answer questions	Cross-sectional	Members of cognitive function and Ageing study previously approached re brain donation	Dementia	594	Combined potential donor and NOK
**Boise, 2017, USA [34]**	Written surveys / cohort study	Cross-sectional	Volunteers registered with NIH-funded Alzheimer’s Disease Centres—African American, Caucasian, Asian and Latino participants	Alzheimer’s disease / dementia	479	Potential donors (Likely)
						Potential donors (Unlikely)
**Sundqvist, 2012, Australia [35]**	Written questionnaire / cohort study	Cross-sectional	NOK who have previously provided consent on the day of autopsy	Alcoholism and psychiatric illness	50	Bereaved NOK
**Garrick, 2009, Australia [36]**	Analysis of phone conversations	Cross-sectional	NOK providing consent for brain donation	Alcoholism and psychiatric illness	200	Bereaved NOK
**Zhang, 2020, China [37]**	Cross-sectional, population based study / in person surveys	Cross-sectional	Randomly sampled community members	Donation for anatomy teaching as well as research	1400	community
**Lerpiniere, 2013 UK [38]**	Community based study	Cross-sectional	Donors and NOK who have experienced a ICH	Intracerebral haemorrhage (ICH)	185	Combined potential donor and NOK
**Millar, 2008, UK [39]**	Community based study / questionnaire provided 6–9 months post donation	Qualitative	NOK following a sudden death	Nonspecific (coronial subjects)	111	Bereaved NOK
**Boise, 2017, USA [34]**	Focus groups with semi-structured interviews	Qualitative	Volunteers registered with Alzheimer’s Disease Centres—African American, Caucasian, Asian and Latino participants	Alzheimer’s disease / dementia	61	Potential donors
					34	NOK
**Bilbrey, 2018, USA [15]**	Focus groups and Online surveys	Qualitative	HCPs	Alzheimer’s disease	10	HCPs
			Community members		42	Community
**Austrom, 2011, USA [40]**	Focus groups with semi-structured interviews	Qualitative	Caregivers of persons with frontotemporal dementia	Frontotemporal dementia	30	NOK
**Chan, 2020, Singapore [41]**	Questionnaire based study	Cross-sectional	People with neurodegenerative disease	Neurodegenerative disease and non-neurodegenerative disorders	122	Potential donors (neurodegenerative)
			People with non-neurodegenerative disease		65	Potential donors (non-neurodegenerative)
**Montoya, 2020, USA [42]**	semi-structured focus group interviews. Sub-study of larger qualitative study on public health messaging	Qualitative	Latino children of parents with Alzheimer’s disease and related dementia	Alzheimer’s disease and dementia	15	NOK
**Lambe, 2011, USA [43]**	Focus groups	Qualitative	African American Elders consented to an Alzheimer’s research registry	Alzheimer’s disease	15	Potential donors
**Akinyemi, 2019, Nigeria [44]**	Semi-structured face to face interview	Cross-sectional	Individuals attending neurology, psychiatry and geriatrics outpatient clinic	Outpatients (neurology, psychiatry and geriatrics)	412	Potential donors
**Harris, 2013, UK [45]**	Interviews on a theoretically sampled population	Qualitative	Patients recruited through PINE study (ongoing incidence and long-term follow-up Parkinson’s study)	Parkinson’s and control	19	Potential donors
**Khan, 2019 Singapore [46]**	Questionnaire	Cross-sectional	Parkinson’s disease patients attending neurology clinic at National Neuroscience Institute	Parkinson’s Disease	105	Potential donors
**Robertson, 2021, Australia [12]**	Written surveys	Qualitative	Parents of children who have consented to post-mortem tumour donation	Paediatric glioma	11	Bereaved NOK (Parents)
**Siminoff, 2021, USA [47]**	semi-structured telephone interviews	Cross Sectional, analytical study	Family decision makers. results not stratified by actual vs hypothetical	Pan-disease	77	NOK not willing to donate
					307	NOK willing to donate
**Glover, 2020, USA [48]**	Qualitative focus groups with semi-structured guides	Qualitative	African Americans	Alzheimer’s disease related dementias	8	Donors (consented)
			Latino Americans		6	Donors (consented)
			Caucasian, low income, Americans		8	Donors (consented)
**Moules, 2021, Canada [11]**	Unstructured in person interviews	Qualitative	Parents of children who have died and donated their tumour to the tumour bank.	Paediatric glioma	5	Bereaved NOK (parents)
**Padoan, 2021, Brazil [49]**	Qualitative interviews either by phone or face to face	Qualitative	Family members approached to consent to brain donation following a suicide	Suicide	41	Bereaved NOK
**Saranza, 2021, Canada [50]**	Anonymised survey questionnaires	Cross-sectional	Patients with a diagnosis of atypical Parkinson’s	Parkinson’s disease	90	Potential donors
**Morlett Paredes, 2022, USA [55]**	Semi-structured Interviews	Qualitative	Latino Persons with Alzheimer’s disease and related dementia	Alzheimer’s disease and related dementia	40	Potential donors
**Singh, 2022, Ghana and Nigeria [51]**	Questionnaires	Cross-sectional	Members of the Stroke Investigative Research and Education Network (survivors, caregivers and healthy controls)	Stroke	1015	Potential donors and NOK combined

### Quality appraisal of studies

Table 2 documents the output of the quality appraisal for each of the studies presenting cross sectional quantitative data. Table 3 contains quality appraisal results from those with primarily qualitative data.

**Table 2:** Quality appraisal for cross-sectional studies.

Author and Year	Inc Criteria defined	Clear subject & setting	Exposure valid and reliable	Objective & Standard Measure	Confounding factors identified	Confounding factors stated	Measure of outcome	Statistical method
Bidaut-Russell 1991 [20]	Y	Y	Y	N	Y	Y	Unclear	Unclear
Shaw 2012 [21]	Y	Y	Y	N	Y	Y	Unclear	Y
Wu 2012 [22]	Y	Y	NA	N	Y	Y	Y	Y
Darnell 2011 [23]	Y	Y	Y	N	Y	Y	Y	Y
Fonseca 2015 [24]	Y	Y	Y	N	Y	Y	Unclear	Y
Glaw 2009 [13]	Y	Y	Y	N	Y	Y	Y	Y
Azizi 2006 [25]	Y	Y	Y	N	Y	Y	Y	Y
Schnieders 2013 [26]	Y	Y	NA	N	Y	Y	Y	Unclear
Garrick 2006 [52]	NA	Y	Y	N	Y	Y	Y	Y
Kuta 2011 [28]	Y	Y	Y	N	Y	Y	Y	Y
Jefferson 2011 [53]	Y	Y	Y	N	Y	Y	Y	Y
Le Bouc 2016 [30]	N	Y	Y	N	Unclear	N	Y	Y
Angelini 2011 [32]	Y	Y	Y	N	Y	Y	Unclear	Unclear
Stevens 1998 [33]	Y	Y	Y	N	Y	Y	Y	Y
Boise 2017 [34]	Y	Y	NA	N	Y	Y	Y	Y
Sundqvist 2012 [35]	Y	Y	Y	N	Y	NA	Y	Y
Garrick 2009 [36]	Y	Y	Y	N	Y	Y	Y	Y
Zhang 2020 [37]	Y	Y	NA	N	Y	Y	Y	Y
Lerpiniere 2013 [38]	Y	Y	NA	N	Y	Y	Y	Y
Chan 2020 [41]	Y	Y	NA	N	Y	Y	Y	Y
Akinyemi 2019 [54]	Y	Y	NA	N	Y	Y	Y	Y
Khan 2019 [46]	Y	Y	NA	N	Y	Y	Y	Y
Siminoff 2021 [47]	Unclear	Y	NA	N	Y	Y	Y	Y
Saranza 2021 [50]	Y	Y	NA	N	Y	Y	Y	Y
**Singh 2022 [51]**	Y	Y	Y	N	Y	Y	Y	Y

Criteria 1: Inclusion criteria clearly defined, Criteria 2 Study subjects and setting described, Criteria 3: Exposure measured in a valid and reliable way, Criteria 4: Objective and standard criteria for measurement, Criteria 5: Confounding factors identified, Criteria 6: Strategies or confounding factors stated, Criteria 7: Outcomes measured in a reliable way/clear analysis, Criteria 8: Appropriate statistical method.

None of the studies presenting cross-sectional data met all 8 criteria, with no study able to employ a standard criterion for measurement. Measurement of exposure was problematic for many studies in that participant exposure and experience of brain donation varied widely. Some studies were able to categorically include participants who had been directly involved in either consenting to a brain donation study or had supported a loved one to donate, while others were collected data from participants based on theoretical scenarios.

One study, Le Bouc 2016, did not identify confounding factors within the data, while three studies Bidaut-Russell 1991, Schnieders 2013 and Angelini 2011 did not clearly include details of their statistical methodology. In two studies, Le Bouc 2016 and Siminoff 2021, inclusion criteria were not outlined or unclear however all provided a clear description of subjects and settings to ensure contextual representation of the data.

**Table 3:** Quality appraisal results from those with primarily qualitative data.

Author and Year	Congruity between;					Awareness of researcher	Influence of researcher	Participant voices	Ethical approval	Logical conclusions
	Philosophy and method	Method and question	Method & data collection	Method, data & analysis	Method and results					
Eatough 2012 [16]	Y	Y	Y	Y	Y	Unclear	Y	Y	Y	y
Millar 2007 [29]	Unclear	Y	Y	N	N	N	Y	Y	Y	Y
Padoan 2017 [31]	Y	Y	Y	Y	Y	N	Y	Y	Y	Y
Millar 2008 [39]	Unclear	Y	Unclear	Unclear	Unclear	N	Y	Y	Y	Y
Boise 2017 [55]	Y	Y	Y	Y	Y	N	N	Y	N	Y
Bilbrey 2018 [15]	Y	Y	Y	Y	Y	N	Y	Y	N	Y
Austrom 2011 [40]	Y	Y	Y	Y	Y	N	N	Y	N	Y
Montoya 2020 [42]	Y	Y	Y	Y	Y	N	N	Y	Y	Y
Lambe 2011 [43]	Y	Y	Y	Y	Y	Y	N	Y	Y	Y
Harris 2013 [45]	Y	Y	Y	Y	Y	N	Y	Y	Y	Y
Robertson 2021 [12]	Y	Y	Y	Y	Y	N	N	Y	Y	Y
Glover 2020 [48]	Y	Y	Y	Y	Y	N	N	Y	Y	Y
Moules 2021 [11]	Y	Y	Y	Y	Y	Y	Y	Y	Y	Y
Padoan 2021 [49]	Y	Y	Y	Y	Y	N	Y	Y	Y	Y
Morlett Paredes 2022 [55]	Y	Y	Y	Y	Y	Y	Unclear	Y	Y	Y

Criteria 1: Congruity between the stated philosophical perspective and research methodology, Criteria 2: Congruity between the research methodology and the research question/objectives, Criteria 3: Congruity between the research methodology and the methods used to collect the data, Criteria 4: Congruity between the research methodology and the representation and analysis of data, Criteria 5: Congruity between the research methodology and the representation and interpretation of results, Criteria 6: A statement locating the researcher culturally or theoretically, Criteria 7: Is the influence of the researcher on the research and vice-versa addressed?, Criteria 8: Are the participants and their voices adequately represented, Criteria 9: Is there evidence of ethical approval by and appropriate body, Criteria 10: Do conclusions flow from the analysis and or interpretation of the data?

Of the 10 criteria for qualitative studies, 3 were consistently met by all studies: Congruity between research methodology and objectives, adequate representation of participant voices and appropriate conclusions drawn from the interpretation of the data. Of the criteria, a statement locating the researcher culturally or theoretically and consideration of the influence of the researcher on the research were the two requirements most frequently unaddressed with only Moules 2021 and Lambe 2011 providing reference to the role of the researcher and the context or lens through which the researcher was working. Morlett Paredes 2022 did locate the researcher culturally and cite the presence of bi-lingual and bi-cultural researchers, but did not explicitly discuss researcher influence.

Subsequent analysis of the data yielded the commonality with which certain themes or ideas were presented within the literature. Tables 4 and 5 below describe the themes identified and the specific perceived benefits described in relation to each theme.

**Table 4:** Perceptions of benefit noted in study.

Category	Benefit (Perceived and Actual)	Study Reference
Altruism	Benefit to future generations / someone else / others / community / give hope	[12,13,21,24,26–29,31,33–36,38–41,46,48–51,55,56]
Altruism / Responsibility	Find a cure / save a life / right thing to do	[11,12,31,33,43,51,53,54,56]
Comfort / Consolation	Consolation and comfort to family: meaning in death / aid grief / closure for family	[11,16,28,29,31,35,41,46,49,55,57]
	Legacy / enduring work—living on in donation / ongoing connection	[11,12,16,31,49]
	Comfort knowing body is buried without tumour	[12]
	Pride in relatives participation	[55]
Control / Empowerment	Practical focus in time of grief / control for family / distraction / empowerment / fulfilling wish	[16,25,29,31,39]
	Consistent with organ donor wishes / implies honourable character	[13,16,35,36]
	Avenue for action motivated by family illness	[13,27,49]
	Consolation and comfort to donor / right thing to do / save a life	[33]
Existential	Existential: donor contribution / meaning to death / purpose to suffering (Donor)	[11,12,21,31,35,47]
	Existential: redemption	[46,49]
Information and Education	Confirm diagnosis / answers about death	[16,20,28,29,35,38,40,53]
	Education for communities / participate in important dialogue (overcome prejudice/ignorance)	[21,48]
	Access to accurate and honest information	[27]
Instigate Change	Cultural change—change funeral belief	[37]
	Cultural change—overcome mistrust in medical field	[43]
Practicality/pragmatism	Brain is of benefit / give brain a use / avoid ’waste’ / won’t need if dead	[11–13,16,21,24,33,38,39,41,42,44,45,55]
	Benefit to NOK or family (hereditary disease)	[16,20,28,40,41,43,46]
	Help family / future generations of family (not-specifically hereditary)	[26,33,43,45,48]
	Benefit to themselves	[26]
	Repay medical care provided	[38]
Religious	Christian call to altruism and empathy	[56]
Research / Medicine / Science	Aid disease research /scientific research (Development and progress)	[20,24,25,29,31–33,35,37,39–41,43,46,49,51,55,56]
	Benefit to Medicine / improve treatments / improve diagnosis (before death)	[12,13,27,28,31–33,35,37,47,49,51,53,55]
	Benefit / Contribution to science / allows donor to show interest in science (Interest)	[13,16,21,22,24,27,38,56]

**Table 5:** Perceptions of harm noted in study.

Category	Actual / potential / misconception	Harm (Perceived and Actual)	Study Reference
Confusion / Anguish	Actual	Fear that family would not understand change in diagnosis post histological examination	[30]
Cultural	Potential	Fear that people will view me as occultic	[51]
Emotional distress	Potential	Emotional distress for consenting NOK at allowing autopsy / Distress for NOK / Responsibility	[20,28,32,35,36,38–41,45–48,55]
	Potential	Discord in family / objections from other family members	[20,24,25,29,30,33,36–39,43,45,55]
	Potential	Finality / giving up the fight / confront reality / forced to think about things	[11,16,28,29,33,45]
	Potential	Upset by conversation / Negative reaction when spoken about	[21,30,47,48]
	Potential	Conversation is perceived as imminent death	[30,38,40]
	Actual	Brain exceptionalism—taking away who I am	[45,46,55]
	Potential	Anxiety due to rapid autopsy / would it run smoothly	[12,16,48]
	Actual	Distressing to be separated from deceased so quickly	[11,12]
	Potential	Damage to enduring ’image’ of deceased / memory	[11,25]
	Actual	Give up control (NOK) / loss of control	[16]
	Potential	Negatively impact family member with mental illness	[25]
Inappropriate motivations	Potential	Not in alignment with wishes of deceased	[15,21,24,25,29,32,35,36,38,39,42]
	Potential	Request accentuates fear / Mistrust / disrespectful researchers / abuse of power/ guinea pig	[15,23,30,34,42–45,51,55]
	Misconception	Concerns around insufficient donor consent	[30,40]
	Potential	Concerns consenting person will be perceived as having greater interest in research than patient care	[30]
	Potential	Guilt—NOK wanting answers despite contrary to wishes of deceased	[16]
	Potential	Concerns about tissue used for profit	[47]
Intrusion	Potential	Invasion of privacy / confidentiality	[22,47]
Physical harm	Misconception	Disfigurement / ’damage’ to donor	[11,16,20,21,24,30–32,34,41,44–46,49,55,56]
	Actual	Brain won’t be intact / removing body parts / incomplete for funeral / not whole	[21,22,24,33,35,42,44,45]
	Actual	Too invasive / Intrusive / Macabre	[31,38,43,53]
	Potential	Might restrict end of life choices / donation might be forced	[15,23,44,45]
	Misconception	Donor might not really be dead	[22,33,44]
	Misconception	Pain after death	[33]
Practical implications	Misconception	Delay to funeral	[20,24,28,30,41,45,46,49,55]
	Misconception	Family might incur additional costs	[20]
Religious/Spiritual implications	Actual	Contrary to religious beliefs / body should be made whole (specific religious connotation)	[16,20,21,25,28,30,32–34,37,38,40,41,43,44,46,50,51,55,56]
	Actual	Concerns around afterlife	[16,31,33,55]
Suffering	Potential	Further suffering or pain for donor / distress for donor	[20,30,32,46,55]

### Perceived benefit

As described in Table 4, commonly documented perceived benefits were the benefit to future generations, a desire to aid disease/scientific research, the idea that the donation avoids waste, benefit to medicine in terms of improved treatments and diagnosis and the belief that donation will assist the family by bringing consolation, meaning or aid the grief process for family.

Codes relating to altruism, both as a responsibility and in a wider context were frequently reported, as were codes related to specifically benefiting research, medicine and science. These can be combined to create a wider theme of altruism. Within the category of ‘comfort and consolation’ two codes related to the idea of ‘living on’ are noted, suggesting that this is a theme both in the practical sense of an enduring cell line and also in an existential or sense of legacy. Codes related to avoidance of waste can also be considered in conjunction with those concerning honourable character and organ donation suggesting alluding to themes of purpose and meaning making.

### Perceived harm

Of the perceptions of harm, the most commonly documented was a perceived conflict with religious beliefs, that donation would result in damage or disfigurement to the donor, that consent would lead to emotional distress for the NOK at time of autopsy, that consent could lead to discord or objections within the family and that donation would not align with the wishes of the deceased. Codes relating to emotional harm were commonly recorded. The majority of perceived harms were noted to be potential harms or misconceptions and this was true for codes relating to emotional distress, inappropriate motivations, physical harm, suffering. These codes hold relevance for discussions of awareness, education and overcoming cultural barriers.

### Inter-group comparisons

#### Paediatric donations compared with wider cohort

Of the 3 studies that investigated the experiences of parents of paediatric brain tumour patients, Angelini 2011, Robertson 2021 and Moules 2021, some the perceptions of benefit were largely aligned with that of the overall cohort–excepting parents’ reported belief that brain donation would provide comfort to them through knowing the child was buried without the tumour. Perceptions of harm were largely aligned with those seen in the wider cohort with one exception, that both Robertson 2021 and Moules 2021 captured insights that parents felt it distressing to be separated from the deceased so quickly, a perception not noted elsewhere in the wider/adult dataset.

#### Health Care Providers (HCPs) compared with wider cohort

There were no data in any of the identified studies relating to perceptions of benefit from HCPs however there were two studies, Le Bouc 2016 and Bilbrey 2018, exploring barriers to HCP engagement and the concerns of HCPs towards brain donation. Within these studies there were two insights unique to this group identified by Le Bouc 2016; The concern that the person obtaining consent may be perceived as having a greater interest in research than in patient care and secondly the fear that should the post-mortem lead to a change in diagnosis this would cause confusion for the family.

#### Next of Kin prior to donation compared to bereaved Next of Kin

A comparative analysis of studies including NOK prior to donation and those who included NOK who were bereaved lead to some clear distinctions in perceptions of both benefit and harm. One study, Bidaut-Russell 1991, combined NOK responses from both those who were bereaved and those interviewed prior to donation; and four studies, Padoan 2017, Stevens 1998, Lerpiniere 2013 and Singh 2022, combined NOK responses with those of potential donors. All five of these studies were excluded from the sub-group analysis as it was not possible to distinguish subgroup (donors or next of kin) attribution.

Of the 8 studies with data specifically collected from bereaved adult NOK (Eatough 2012, Fonseca 2015, Azizi 2006, Millar 2007, Sundqvist 2012, Garrick 2009, Millar 2008 and Padoan 2021), 4 studies [16,35,39,49] indicated that NOK in this state see brain donation as a practical focus in their time of grief, providing a sense of control, a distraction and an empowering process enabling them to fulfil a wish. This insight was not noted as a potential benefit in the studies including NOK prior to donation. Similarly, bereaved NOK perceived brain donation as a source of legacy allowing them to remain connected to the deceased, an insight that was not conveyed by the NOK interviewed prior to donation. Other perceptions of benefit unique to bereaved NOK included that donation implied donor had an honourable character, that it was the right thing to do, that it provided a source of redemption for the donor and that it afforded the NOK comfort knowing the body was buried without the tumour.

Seven studies included NOK prior to donation exclusively (Wu 2012, Fonseca 2015, Boise 2017, Austrom 2011, Montoya 2020, Le Bouc 2017 and Siminoff 2021) and in Boise 2017 it was expressed that brain donation led to a sense of pride for NOK when reflecting on the commitment to donate and the altruistic intent of their loved one. This was not explicitly noted in the data obtained from bereaved NOK, though may be implied through the references to honourable character [11,35,36].

The variation between bereaved NOK and those prior to donation was much greater with respect to perceptions of harm. Bereaved next of kin referenced concerns around potential discord in family [24,25,29,36,39], anxiety due to autopsy [16], damage to image of the deceased [25] as well as guilt for consenting for ‘selfish’ reasons [16]. Alternatively, NOK prior to donation referenced potential harms such as further pain or suffering for the donor [30,55], abuse of privacy [22,47], concerns around tissue used for profit [47] and concern that the consent conversation is symbolic of death being imminent [40].

## Discussion

### Quality appraisal

The data are heterogenous with regard to setting, context, methodology and personal experience. This presented numerous challenges for quality appraisal. None of the cross-sectional studies was able to apply a standard and objective form of measurement, however this is due to an absence of validated tools within the literature. However, when studies present personal insights and depictions of human experience in an emerging area it can be argued that standardised measurement may decrease the richness of the data [58]. It is likely this will be addressed in coming years as work in the area continues. For example, Glaw *et al* 2009 [13] reported a previously published tool and Garrick *et al* 2006 [27] piloted a measure.

Surprisingly only two studies assessed with the qualitative quality appraisal tool demonstrated awareness of the role of the researcher. Whether this is a deliberate choice to remove researcher subjectivity, is unclear. Given the well documented value of reflexivity [59], and the distinctly human experience of the subject area, it would be encouraging to see a greater representation of the role of the researcher in future studies.

### Perceptions of benefit and harm–variables for consideration

With respect to broad perceptions of benefit, altruistic outcomes were predominant in the literature. We identified two clear altruistic categories, however many others such as ‘benefit to health and medicine’ or ‘helping future generations of family’ can also be seen as having altruistic intent leading to a prevailing theme of altruism. Considerable work has been conducted into the theories of and role of altruism with respect to post-mortem tissue donations, so much so that Quinn et al 2013 suggests it as intrinsic to recruitment strategies [60]. There are three key theories of altruism pertaining to biological donations; pure altruism which refers to donation as a gesture of good will, reciprocal altruism [61] which entails expectations of return for either the donor or the family, and empathy-induced altruism where a shared experience or improvement to the welfare of a group/culture is anticipated [62]. All three of these theories can be identified within the data relating to brain donation however it could be argued that reciprocal and empathy-induced underpin the vast majority of codes identified, particularly with respect to benefit to family, future generations and to those themes categorised as ‘education/information’ and ‘instigating change’.

Interestingly, it could be argued that the data regarding the need to ‘repay’ medical care illustrates a somewhat inverted form of reciprocal altruism whereby donation is an act of reciprocity rather than carried out with anticipated reciprocity. Reciprocal altruism is fraught with ethical pitfalls and as Quinn identifies, can lead to a “web of mutual obligations” whereby a patient may feel obligated to donate due to the efforts of the medical team but also assume that their donation renders researchers obligated to deliver a cure or provide higher level care to surviving family [60]. Perceptions of benefit that are linked with reciprocal altruism while valid and empowering for donors, require careful consideration. Care should be taken to manage expectations during consent to ensure psychosocial benefits to the participant are maximised and unwarranted concerns about potential treatment are not part of the decision-making process.

Both in the oncology and non-oncology settings we coded data relating to ‘living on’ through a donation [11,12,16,31,49]. In a non-oncology space, where sense of self could be clearly associated with brain tissue this appears rational–particularly in light of brain exceptionalism, the notion that the brain holds special significance to sense of self [63]. In an oncology space, however, and particularly where studies may include populations where only malignant tumour was removed, these insights become even more interesting. This is well demonstrated through the unique insights captured by the paediatric oncology studies included in this review. In the work of Moules et al on paediatric cell lines, parents reported being comforted by the knowledge that their child was ‘living on’ in the tumour cell line [11] referencing a sense of connection to the culture. This is a sentiment echoed in numerous other studies, both oncology and non-oncology such as Padoan et al [31] and ties in with broad references throughout the wider brain donation literature to living on and legacy being a primary benefit [11,12,16,31,49].

Somewhat of a misalignment occurs, however, when one juxtaposes the idea of the child ‘living on’ through cell line development with the notion that parents are comforted knowing their child was buried ‘without their tumour’ [12]. This sentiment resonates with the experiences of our study team who operate in a brain cancer context, suggesting that the notion of removing something negative is equally powerful in comforting the bereaved as are promises of creating a legacy. Whether this dichotomy of ‘something antagonistic to excise’ vs ‘something protagonist to treasure and maintain’ is the result of a selective understanding/rationalisation on the part of the individual, or variations in the language used by the consenting team is unclear. This may also be due to variations in tissue collection, namely whether the brain as a whole is removed or a partial tumour. Further work to understand the impact of this variable is required to fully characterise the human experience of brain donation in the paediatric setting but is outside the scope of this review.

While cited as a benefit, recurring references to ‘avoiding waste’[11–13,16,21,24,33,38,39,41,42,44,45,55] are interesting when one considers the harm that could be caused by refusing brain donation. While there are exceptions, broadly speaking, people with a metastatic cancer diagnosis or who have received a systemic form of chemotherapy are unable to donate tissues for transplantation purposes in Australia [64]. Donor families report participating in organ donations can be a resolving factor in grief [65] and it can serve as a way of giving back for the greater good or avoiding waste for donors [66]. If organ donation is perceived as a means of producing hope or ‘good’ from tragedy [67] then it stands to reason that the ineligibility of people with a cancer diagnosis–including those who have consciously consented to organ donor registries–adds to the disappointments felt by patients and their families. In light of this, it’s clear that for many, brain donation for research provides a means from which to restore purpose and meaning and ensure that ‘good’ can come from suffering even when organ donation for transplantation is no longer an option. This is particularly well illustrated by Eatough *et al* whereby a respondent was comforted by references to ‘harvesting’ brain tissue which resonated with her in terms of her life continuing to be ‘fruitful’ following her death [16]. Further support exists in the paediatric setting with recent discussions proposing that brain donation may actually be seen as a palliative intervention due to the emphasis on legacy and meaning-making [68].

It remains to be seen as to whether the sense of empowerment or comfort is greater for those who have previously been refused organ donation for transplantation due to systemic chemotherapy, such as in a brain cancer diagnosis. That said, it could be suggested by the frequent references to organ donation and association of organ donor status with honourable character [13,16,35,36] that for a committed organ donor who is ineligible to donate, the opportunity to participate in a brain donation study directly influences the human experience of participating–restoring purpose and meaning, particularly for altruistically minded persons.

The data indicate that the primary perception of harm associated with brain donation is an incompatibility with religious beliefs or teachings. The work by Wu et al work explored this in detail with respect to Confucian teachings in Taiwanese culture and the moment the soul is believed to leave the brain [22]. While there may be concerns in religious communities such as Confucians, Shintoists and Orthodox Jews, along with concerns raised by South Asian Islamic scholars, it has been cited that no known religious doctrine formally objects to or prohibits organ donation [69]. The body of evidence examining this subject is almost exclusively in a transplantation context however the findings are transferable when the broad understanding is that consent to organ donation or receipt is not explicitly discussed in sacred texts and therefore left to the conscience of the individual. This has been outlined in the Jehovah Witness faith, a group typically associated with objections to blood transfusions or biological medical interventions [70]. It appears that in many instances the concerns of religious councils against organ donation lie in broader concerns regarding the definition of death and the acceptance of cardio-cessation but not brain death as a legitimate death [71]. This serves as a differentiating factor for post-mortem donations and suggests that those with concerns of post-mortem donations may benefit from improved communication with, and facilitated education from, religious and spiritual leaders.

Many of the perceived harms of brain donation are potential misconceptions with practicalities such as ‘delay to funeral’, fears of ‘disfigurement to donor’ and the concern that the ‘donor might not be dead’ clear examples. The nature of rapid autopsy ensures no delay to funeral arrangements with processes typically completed within 24 hours and the nature of the procedure itself ensures donors are fit for open casket or to be viewed by loved ones with little to no obvious signs of autopsy or physical trauma. Likewise concerns around ‘awake’ or ‘live’ autopsy can be allayed with sufficient education and communication around the necessity for a death certificate and thorough examination by a medical professional. The persistence of these views is unsurprising when one considers Khuta’s data indicating that in a USA movement disorders clinic only 78% of participants had heard of brain donation and only 7.5% of those who were familiar with the concept had received educational material from a health care provider [28]. This indicates the need for improved communications underpinned by extensive stakeholder engagement to ensure educational interventions are appropriate. For example, in the work of Boise [55] and Lambe [43], the perceived harm of inappropriate use of donated tissue is likely grounded in historical cultural abuses and requires far greater consideration than concerns of ‘disfigurement’ identified by Chan et al [41] which can be quite simply allayed.

Future work in the area should also consider the potential harms such as ‘privacy’, ‘confidentiality’ and a ‘restriction of end-of-life choices’. While these harms are not outside the realm of possibility, informed consent processes should detail the safeguards and measures employed by biobanking facilities to mitigate the risks of privacy and confidentiality breaches. Likewise, while the donation process does have logistical and feasibility requirements that could potentially impact end of life decisions, we have shown previously that through planning and informal systems reform, rapid brain tissue donation can be facilitated in a manner consistent with the end of life wishes of the donor–despite geographical or logistical challenges [72]. Crucial to facilitating this is a multi-disciplinary clinical support team, united in supporting the patient to donate once consented however the data collected in this review suggests this may be an enduring challenge for brain donation teams.

This is particularly relevant when one considers the insights unique to health care providers, namely the concern that raising the topic of donation may lead patients to view clinicians as having a greater interest in research than in patient care. Within the wider literature search, no data was obtained on health care providers perceptions of benefit. This is reinforced by Le Bouc et al’s findings that the major limiting factor in consent to brain donation is clinicians being unwilling to raise the idea [30]. Whether health care providers do not perceive value in brain donation cannot be answered in the absence of data, however we can infer from broader biobanking data that in many cases health care providers do perceive value, but feel ill equipped to enter into conversations regarding consent [73]. This finding appears relevant and aligns with concerns that an improper or mismanaged conversation could damage the doctor patient relationship and lead to further harm, again reinforcing the importance of education–not only for patients and families but for health care providers. As such, it can be inferred that the relationship between donor and health care provider, and the degree to which the health care provider is supported and equipped to facilitate brain donation conversations is highly influential for the lived experience of brain donation.

### Limitations

Heterogeneity in study type and population is a limitation to this study, introducing confounding variables such as donor cognitive ability, level of dependence on carers, frequency of interaction with the health service, duration of illness and imminence of mortality. Geographic variability is also highly pertinent and a considerable limitation to this study when one considers the diversity in international health care systems and the variability in HCP relationships to patient, resource scarcity/availability and integrated psychosocial support.

## Conclusions

A growing body of literature documents perceptions of benefit and harm relating to post-mortem brain donation for research purposes. The perceptions of benefit include hope, comfort and empowerment for donors and their families and in many cases the perceptions of harm are concerns that can be addressed though education, communication and informed consent that involves both donors and their families. The nature of tissue collection, the status of the donor as a registered organ donor, the cultural background of the individuals concerned and the experience of the Health Care Provider are all factors that influence the human experience of brain donation and many require additional consideration and exploration to ensure they are appropriately managed and addressed. Future work in disease-specific populations is needed to provide a greater understanding of the experiences of individuals consenting to brain donation. Such insight would ensure value is maximised for both the medical research community, consented donors and next of kin.

## Supporting information

S1 Checklist PRISMA 2009 checklist. (PDF)
